# *TaLAMP1* Plays Key Roles in Plant Architecture and Yield Response to Nitrogen Fertilizer in Wheat

**DOI:** 10.3389/fpls.2020.598015

**Published:** 2021-01-08

**Authors:** Ji Shi, Yiping Tong

**Affiliations:** ^1^State Key Laboratory of Plant Cell and Chromosome Engineering, Institute of Genetics and Developmental Biology, Chinese Academy of Sciences, Beijing, China; ^2^College of Advanced Agricultural Sciences, University of Chinese Academy of Sciences, Beijing, China; ^3^The Innovative Academy of Seed Design, Chinese Academy of Sciences, Beijing, China

**Keywords:** *Triticum aestivum* L, LAMP1, agronomic trait, nitrogen uptake, grain nitrogen concentration, nitrogen fertilizer response, plant architecture

## Abstract

Understanding the molecular mechanisms in wheat response to nitrogen (N) fertilizer will help us to breed wheat varieties with improved yield and N use efficiency. Here, we cloned *TaLAMP1-3A*, *-3B*, and *-3D*, which were upregulated in roots and shoots of wheat by low N availability. In a hydroponic culture, lateral root length and N uptake were decreased in both overexpression and knockdown of *TaLAMP1* at the seedling stage. In the field experiment with normal N supply, the grain yield of overexpression of *TaLAMP1-3B* is significantly reduced (14.5%), and the knockdown of *TaLAMP1* was significantly reduced (15.5%). The grain number per spike of overexpression of *TaLAMP1-3B* was significantly increased (7.2%), but the spike number was significantly reduced (19.2%) compared with wild type (WT), although the grain number per spike of knockdown of *TaLAMP1* was significantly decreased (15.3%), with no difference in the spike number compared with WT. Combined with the agronomic data from the field experiment of normal N and low N, both overexpression and knockdown of *TaLAMP1* inhibited yield response to N fertilizer. Overexpressing *TaLAMP1-3B* greatly increased grain N concentration with no significant detrimental effect on grain yield under low N conditions; TaLAMP1-3 *B* is therefore valuable in engineering wheat for low input agriculture. These results suggested that *TaLAMP1* is critical for wheat adaptation to N availability and in shaping plant architecture by regulating spike number per plant and grain number per spike. Optimizing *TaLAMP1* expression may facilitate wheat breeding with improved yield, grain N concentration, and yield responses to N fertilizer.

## Introduction

Nitrogen (N) is the most important macronutrient required for crop growth and development. Global yield variability is heavily controlled by fertilizer use, irrigation, and climate (Mueller et al., 2012). Agriculture is facing great challenges to ensure global food security by increasing yields while reducing environmental costs (Chen et al., 2014). Crops with a higher N use efficiency (NUE) is essential for the sustainability of agriculture, which could minimize the loss of N and reduce environmental pollution. NUE is defined as the grain yield produced per unit of N available in the soils and can be divided into two plant physiological components: N uptake efficiency and N utilization efficiency (Kant et al., 2011; Xu et al., 2012). The plant has two general stages for N use. During biomass formation, there is the amount of N uptake, storage, and assimilation into amino acids and other important nitrogenous compounds. During the grain filling, N is partitioned to seeds, resulting in final yield. In the processes of N uptake and assimilation, the function of several structural genes has been studied extensively in the past decade (Lea and Azevedo, 2006; Xu et al., 2012; Krapp, 2015). A variety of nitrate and ammonium transporters function in N absorption by roots (Crawford and Glass, 1998; Forde, 2000; Howitt and Udvardi, 2000; O’Brien et al., 2016) and a number of enzymes for assimilation and transfer of the absorbed N into amino acids and other compounds, such as nitrate reductase, glutamine synthetase, and glutamate synthase (Campbell, 1988; Lam et al., 1996; Näsholm et al., 2009).

It is important to understand the N fertilizer response of crops in improving NUE either by increasing yield under existing levels of N supply or by maintaining yield by decreasing N levels (Kant et al., 2011). Genome-scale gene expression has been successfully used to identify key genes in mediating NUE. An early nodulin (*ENOD93*) gene was identified by transcriptional profiling experiment, and the function in regulating NUE was evaluated by transgenic approach, overexpressing the *OsENOD93-1* gene increased shoot dry biomass and grain yield of transgenic rice plants (Bi et al., 2009). In microarray analysis, *TaNFYA-B1* was found to be upregulated by low N and low phosphorus (P) availabilities, and overexpression of *TaNFYA-B1* increased grain yield and grain N concentration under low N and P supply levels in a field experiment (Qu et al., 2015). A NAC transcription factor *TaNAC2-5A* was upregulated under low N conditions; overexpression of *TaNAC2-5A* increases shoot N accumulation and grain yield, with higher N harvest index (He et al., 2015).

Map-based cloning also identified several genes important for NUE and yield performance. *OsNRT1.1B* diverges between *indica* and *japonica* of cultivated rice; *OsNRT1.1B*-*indica* variation was associated with enhanced nitrate uptake and root-to-shoot transport and upregulated expression of nitrate-responsive genes. In the field experiment of near-isogenic and transgenic lines, the *OsNRT1.1B*-indica allele has significantly higher grain yield and NUE compared with the lines without the allele (Hu et al., 2015). *OsDEP1* was a coincidence with *qNGR9*, a major quantitative trait locus for N-regulated plant growth response; the different alleles of *OsDEP1* have different N response, rice carrying with *dep1-1* allele could improve harvest index and grain yield at moderate levels of N fertilization (Huang et al., 2009; Sun et al., 2014). Recently a genome-wide association analysis has identified the NAC transcription factor *OsNAC42* and its downstream nitrate transporter *OsNPF6.1*, which are critical for the response of panicle number to N availability; the elite haplotypes of these two genes greatly increased NUE and yield in rice (Tang et al., 2019). These results show that the gene involved in the response of yield components to N availability is critical for NUE and yield.

Plant hormones are important for plant development and response to environmental cues, e.g., the response of plants to nutrient availability (Rubio et al., 2008; Krouk et al., 2011). The plasticity of root architecture in response to N availability largely determines N acquisition efficiency. The auxin biosynthesis *Tryptophan Aminotransferase-Related* genes *AtTAR2* in *Arabidopsis* and *TaTAR2* genes in wheat plays critical roles in regulating lateral root development under low N treatment (Ma et al., 2014; Shao et al., 2017). Overexpression of *TaTAR2.1-3A* increased grain yield under both low and high N conditions (Shao et al., 2017). N fertilizer is known to upregulate the biosynthesis of cytokinin, which is coordinating shoot and root development, and thus spatiotemporal regulation of cytokinin would benefit crop traits of yield and improve nitrogen use efficiency (Gu et al., 2018). Cytokinin levels are regulated by a balance between biosynthesis (IPT) and degradation, decreased expression of CKX, and enhanced IPT activity resulting in enhancing seed size. During senescence, the levels of active cytokinin’s decrease, with premature senescence leading to a decrease in yield (Jameson and Song, 2016).

Glutamate carboxypeptidase II (GCPII) catalyzes the hydrolysis of N-acetylaspartylglutamate to glutamate and N-acetylaspartate (Rojas et al., 2002; Mesters et al., 2006). The plant putative GCPIIs have a high degree of conservation with the animal GCPIIs, possessing the N-terminal membrane-spanning domain, conserved zinc residues, and catalytic residues (Poretska et al., 2016). However, the biochemical function of the plant GCPIIs is currently unknown. The *Altered Meristem Program 1* (*AMP1*) in *Arabidopsis* encodes a putative glutamate carboxypeptidase and has significant similarity with mammalian N-acetyl-linked acidic dipeptidases (Helliwell et al., 2001). *Arabidopsis amp1* mutant was isolated on the basis of an increased rate of leaf initiation and increased level of cytokinin biosynthesis (Chaudhury et al., 1993; Chin-Atkins et al., 1996; Nogué et al., 2000). Loss-of-function of *AtAMP1* has pleiotropic effects on plant development, including altered shoot apical meristems, increased cell proliferation, polycotyly, constitutive photomorphogenesis, and early flowering time (Chaudhury et al., 1993; Helliwell et al., 2001). The contents of amino acids are changed in *amp1* mutant compared with wild type (WT) (Shi et al., 2013). The pleiotropic phenotypes of the *amp1* mutants may be associated with the critical role of *AtAMP1* in translation inhibition of microRNAs (miRNAs), as *AtAMP1* mediates the activities of multiple miRNAs in reducing the protein levels of their target genes (Li et al., 2013). *AtAMP1* and its paralog *LIKE AMP1* (*AtLAMP1*) have partially overlapping roles in regulating plant development; AMP1 is an endoplasmic reticulum integral membrane protein that interacts with Argonaute1 and requires miRNA-mediated translation inhibition of target mRNAs but not mRNA cleavage (Li et al., 2013; Huang et al., 2015). *ZmVP8*, the ortholog of *AMP1* in maize (*Zea mays*), modulates meristem development and seed maturation by controlling the accumulation of abscisic acid (Suzuki et al., 2008). *OsPLA3*, the *VP8* orthologs in rice, regulates various developmental processes and cytokinin homeostasis (Kawakatsu et al., 2009). However, it is unclear what primary defect could account for the various aspects of the mutant phenotype of *Arabidopsis*, rice and maize. Despite a wealth of phenotypic data, GCPII function has not been linked to N response, especially in wheat.

Here, we found that *TaLAMP1* is inducible by N starvation in wheat. The altered expression level of *TaLAMP1* influenced the root growth and N uptake at seedling. In the field experiment, both overexpression and knockdown of *TaLAMP1* reduced grain yield and yield response to N fertilizer. We also found that altering *TaLAMP1* expression changed root morphology and plant architecture of wheat plants.

## Materials and Methods

### Plant Materials and Growth Conditions

The wheat (*Triticum aestivum* L.) variety Kenong 199 (KN199) was used to isolate the sequences and evaluate the expression of the *TaLAMP1* genes, and KN199 was used to generate the transgenic lines. The T3 transgenic lines were used in the hydroponic culture. The nutrient solution and growth conditions of the hydroponic culture were described by Shao et al. (2017). Briefly, two N treatments of high N (2-mM N NO_3_^–^) and low N (0.2-mM NO_3_^–^) were set with four replicates for each treatment. CaCl_2_ and KCl were used to balance the calcium and potassium concentrations of the different treatments. The growth chamber was with 20 ± 1°C, 50 to 70% relative humidity, a photon fluence rate of 300-mmol photons m^–2^ s^–1^, and 16 h day/8 h night cycle conditions. Five days after germination, wheat seedlings were transferred to plastic boxes containing nutrient solution, and the solution was refreshed every 2 days. After grown for 12 days, the roots and shoots of three plants from three biological replications were collected separately for gene expression analysis. The roots and shoots of four plants from four biological replications were collected separately for measuring shoot dry weight, root dry weight, and root morphological parameters. The root morphological parameters were measured as described previously (Ren et al., 2012). The N concentration in roots and shoots were measured using a semiautomated Kjeldahl method (Kjeltec Auto 1030 Analyzer, Tecator).

The T4 transgenic lines were used in the field experiment in the 2015–2016 wheat growing season, which was conducted at the experimental station of the Institute for Cereal and Oil Crops, Hebei Academy of Agriculture and Forestry Sciences, Hebei Province, China (114.725891 E, 37.948118N). The experiment consisted of high N (225 kg N fertilizer per ha) and low N (94.5 kg N fertilizer per ha) treatments. The N fertilizer was applied as urea, with 2/3 applied before sowing and 1/3 applied at the stem elongation stage. The field experiment followed a randomized block design with four replications. The seeds were manually sown on September 29th of 2015; the plants were harvested in the middle of the following June. Each individual lines in each replication had a 2-m long row with a spacing of 5 cm between plants and 23 cm between rows. Field management followed normal agronomic procedures during the whole growth period. At maturity, 30 representative plants were harvested to measure plant height, biomass yield per plant, spike number per plant, grain yield per plant, grain number per spike, grain weight per spike, harvest index, and N concentration in aerial parts. According to the dry weight of 500 dried grains, a 1,000-grain weight was determined, which was manually counted. During the grain filling stage, we measured Soil Plant Analysis Development (SPAD) values at six time points of the flag leaves. SPAD values were determined quickly using SPAD-502 chlorophyll meter (Zhejiang Top Cloud-Agri Technology Co., Ltd.).

### Vector Construction and Transformation

The overexpression construct harboring only the coding sequence of *TaLAMP1-3B* complementary DNA (cDNA), a 2,910-bp fragment, including the whole coding region, was inserted into the vector pJIT163 under the control of the ubiquitin promoter. This was subsequently transformed into KN199 by particle bombardment, and nine independent transgenic lines were harvested. OE1, OE2, and OE3 were used for phenotypic evaluation. The *TaLAMP1* RNAi construct was generated by insertion of a hairpin sequence containing two 481-bp cDNA inverted repeat fragments targeting the *TaLAMP1* sequence of KN199 into pUBI-RNAi, under the control of the ubiquitin promoter. We got eight independent RNAi transgenic lines and phenotypically evaluated three of the RNAi lines, R1, R2, and R3.

### Quantitative Real-Time PCR

Real-time quantitative PCR was performed using the LightCycler 480 system (Roche, Mannheim, Germany) using the LightCycler 480 SYBR Green I Master Mix (Roche, Mannheim, Germany). Total RNA was extracted with TRIzol reagent (Thermo Fisher Scientific, Waltham, MA, United States). Reverse transcription was performed with the Maxima cDNA Kit with dsDNase (Fisher Scientific Italia, Thermo Fisher Scientific Inc) using 1-μg total RNA. The relative transcript level of each cDNA sample was calculated from triplicates using the formulate 2^–Δ^ ^Ct^ after normalization to *TaActin* (wheat) control. The primers used for qRT-PCR analysis are listed in Supplementary Table 1.

### Subcellular and Tissue Localization

pRI101-35S:TaLAMP1-3B–eGFP was transformed into *Arabidopsis* by dipping method; root tip cells of T2 lines were observed with a Zeiss LSM 710 NLO laser scanning microscope (Zeiss, Germany)^1^ with 488- and 543-nm laser.

### Zeatin Riboside Qualification

The seedlings of KN199 and the T3 transgenic lines *TaLAMP1* were used in the hydroponic culture containing 2.0-mM NO3^–^ for 5 days; the whole seedlings were collected and frozen in liquid nitrogen and ground to a fine powder. To quantify the content of zeatin riboside (ZR), 0.5-g powder of each transgenic line was taken. The methods for extraction and purification of ZR were described previously (Zhang et al., 2016). Our samples were sent to the Phytohormone Research Institute of China Agricultural University for ZR analysis using enzyme-linked immunosorbent assay according to the procedure.

### Nitrate Uptake Assay Using ^15^NO_3_^–^

The seedlings of KN199 and the T3 transgenic lines *TaLAMP1* were grown for 7 days in the nutrient solution containing 2.0-mM NO3^–^ (high N), then transferred to 0.2-mM NO_3_^–^ (low N) for 2 days and resupply to 2.0-mM ^15^NO_3_^–^; roots were collected for ^15^N analysis, as described previously (Chen et al., 2016). The plants were transferred to 0.1-mM CaSO_4_ for 1 min, then to 2.0-mM ^15^NO_3_^–^ nutrient solution with a 99% atom excess of ^15^N for short-term labeling (5 min). At the end of labeling, the roots were washed for 1 min in 0.1-mM CaSO_4_ and quickly separated from the shoots. ^15^N-labeled nutrient solution was made from Ca(NO_3_)_2_ (98 atom% ^15^N, 98%, Sigma-Aldrich Company, United States). Roots samples were dried overnight at 80°C, and the ^15^N content was measured using the Antineutrophil cytoplasmic antibody–multiple sclerosis system (PDZ Europa Ltd). Net ^15^NO_3_^–^ absorption value = DW ^∗^ δ^15^N.

### Statistical Analysis

One-way analysis of variance was performed with SPSS17.0 for Windows (SPSS). Sequence data from this article can be found in the Gramene^2^ data libraries under the following accession numbers: *TaLAMP1-3A*, TraesCS3A02G276800; *TaLAMP1-3B*, TraesCS3B02G311000; *TaLAMP1-3D*, TraesCS3D02G277000; *TaNRT2.1-6B*, TraesCS6B02G044300; *TaNPF6.3-1D* (*NRT1.1B*), TraesCS1D02G214300; *TaNR1-6D*, TraesCS6D02G020700; *TaGS1-6A*, TraesCS6A02G298100; *TaGDH2-2B*, TraesCS2B02G409300.

## Results

### Identification of *TaLAMP1* Genes in Wheat

**FIGURE 1:**
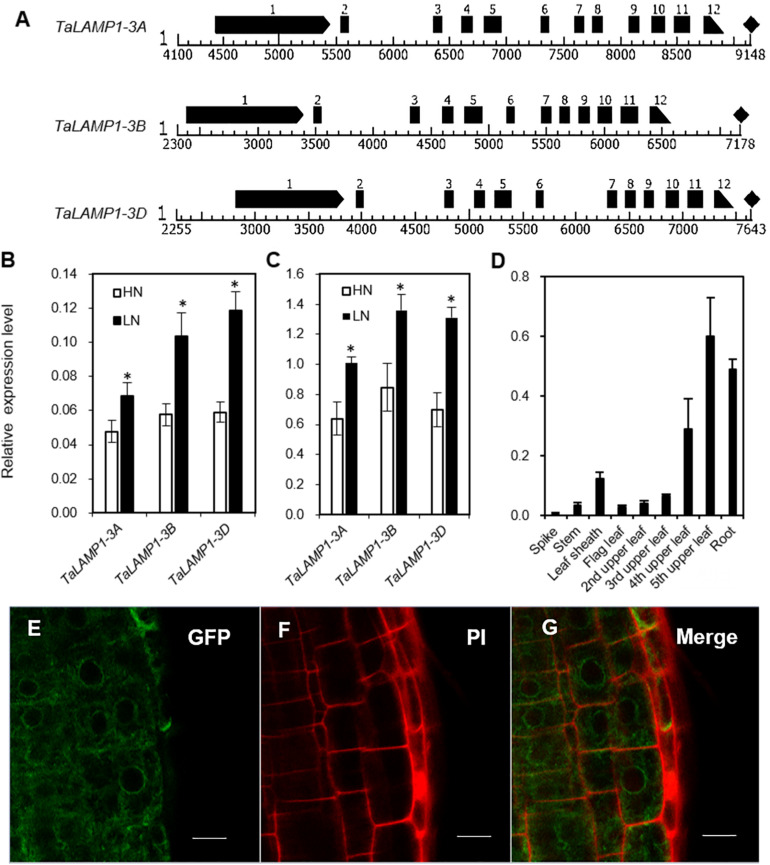
Genomic structure and expression pattern of *TaLAMP1* and subcellular location of TaLAMP1. **(A)** Genomic structure of *TaLAMP1* genes in wheat. Black boxes mean exons of *TaLAMP1*; there are 12 exons in *TaLAMP1-3A*, *3B*, and *3D*. **(B,C)** Expression of *TaLAMP1-3A*, -*3B*, and -*3D* in shoots **(B)** and roots **(C)** of the wheat seedlings under different N supply levels. Seedlings of KN199 were grown for 10 days in the nutrient solution containing 2.0-mM NO_3_^–^ (HN) and 0.2-mM NO_3_^–^ (LN); then, shoots and roots were collected for gene expression analysis. “*” indicates statistically significant differences between HN and LN treament at the *P* < 0.05 level. **(D)** Expression of *TaLAMP1* in different tissues at 14 days post-anthesis (DPA) of the field-grown KN199 plants. Wheat plants were grown under high N conditions; the roots and aerial parts were collected at 14 DPA. Gene expression was normalized using the expression of *TaActin*. Data are mean ± SE of three biological replications. **(E–G)** Root cells of *Arabidopsis* transformed with a *35S:TaLAMP1-3B:GFP* plasmid **(E)**, stained with PI **(F)**, and merged **(G)**. Bars = 10 μm.

**FIGURE 2:**
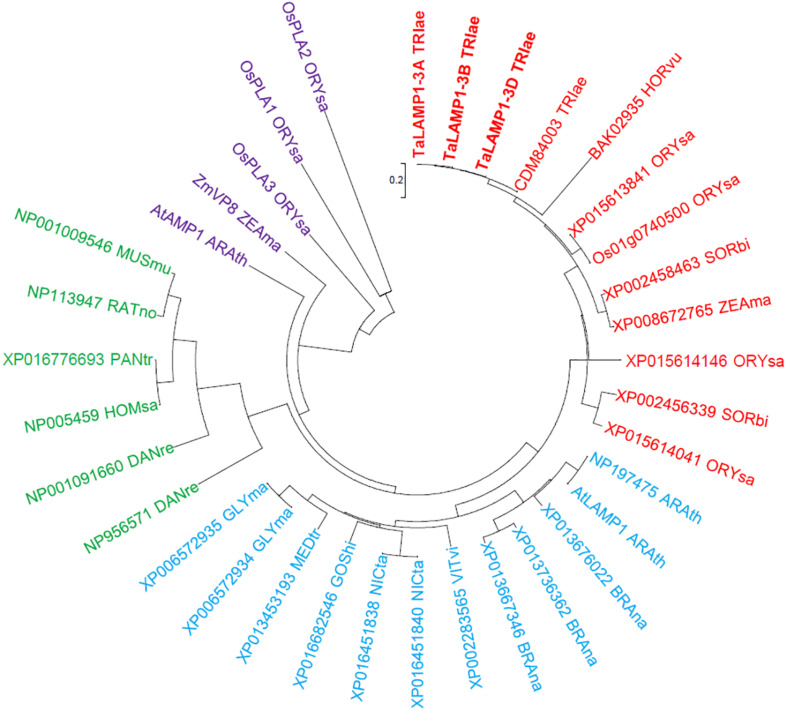
Phylogenetic analysis of GCP II proteins. Neighbor-joining (NJ) method was used to perform a phylogenetic analysis of 35 GCP IIs from 18 species such as *Triticum aestivum* (TRIae), *Hordeum vulgare* (HORvu), *Oryza sativa* (ORYsa), *Sorghum bicolor* (SORbi), *Zea mays* (ZEAma), *Arabidopsis thaliana* (ARAth), *Brassica napus* (BRAna), *Vitis vinifera* (VITvi), *Nicotiana tabacum* (NICta), *Gossypium hirsutum* (GOShi), *Medicago truncatula* (MEDtr), *Danio rerio* (DANre), *Glycine max* (GLYma), *Homo sapiens* (HOMsa), *Pan troglodytes* (PANtr), *Rattus norvegicus* (RATno), and *Mus musculus* (MUSmu). Red, blue, and purple colors highlight the GCPIIs of the plant. Green color highlights the GCPIIs of animals.

Variations in gene expression profiles were analyzed by Affymetrix wheat 61K GeneChip of Wheat variety Kenong 9204 (KN9204) flag leaves in the grain filling stage. The N concentrations in flag leaves were found to increase with N application rates (Supplementary Figures 1A,B). Two probes *Ta.9132.3.S1_s_at* and *Ta.5139.1.S1_at* were found to be upregulated by low N treatment (Supplementary Figures 1C,D). Based on the sequences provided by the Plant Expression Database and the National Center for Biotechnology Information, we isolated the full-length cDNA and genomic DNA sequences of three *TaLAMP1* partial homologs from the winter wheat variety KN199 by rapid amplification of cDNA ends and genomic walking PCR. The coding region of the three *TaLAMP1* genes each consists of 12 exons interrupted by 11 introns (Figure 1A) and encoding a protein of 729 amino acid residues (Supplementary Figure 2). The TaLAMP1s shared 95% amino acid sequence similarity between each other and had three functional domains of peptidases superfamily, zinc peptidases such as superfamily and TFR dimer superfamily (Supplementary Figure 2). A phylogenetic tree was constructed with the full-length putative amino acid sequences of GCPII subfamily members of plants and animals. Phylogenetic assays showed that TaLAMP1-3A, 3B, and 3D clustered in the same clade with AtLAMP1 (Figure 2).

### Expression Pattern of *TaLAMP1* in Wheat

To further evaluate the response patterns of *TaLAMP1* expression to N availability, we analyzed the expression of *TaLAMP1-3A*, *3B*, *3D* in wheat seedlings grown in 2-mM NO_3_^–^ (high N treatment) and 0.2-mM NO_3_^–^ (low N treatment). Compared with high N treatment, the expression of *TaLAMP1* genes was significantly increased in both shoots and roots under low N treatment, with higher expression in roots than in shoots (Figures 1B,C). At 14 days post-anthesis (DPA), *TaLAMP1* transcripts were detected in all the investigated organs, with relatively higher expression in roots and aging leaves and lower expression in spikes, stems, leaf sheaths, and young leaves (Figure 1D). We next investigated the subcellular localization of TaLAMP1-3B and root epidermal cells, and protoplast of wheat confocal microscopy analyses showed that TaLAMP1-3B was cytoplasm localized (Figures 1E–G and Supplementary Figure 3). These results suggested that TaLAMP1 might participate in wheat N response and play a function in both shoots and roots during vegetative and reproductive development.

### *TaLAMP1* Affected Wheat Growth and N Uptake at Seedling

**FIGURE 3:**
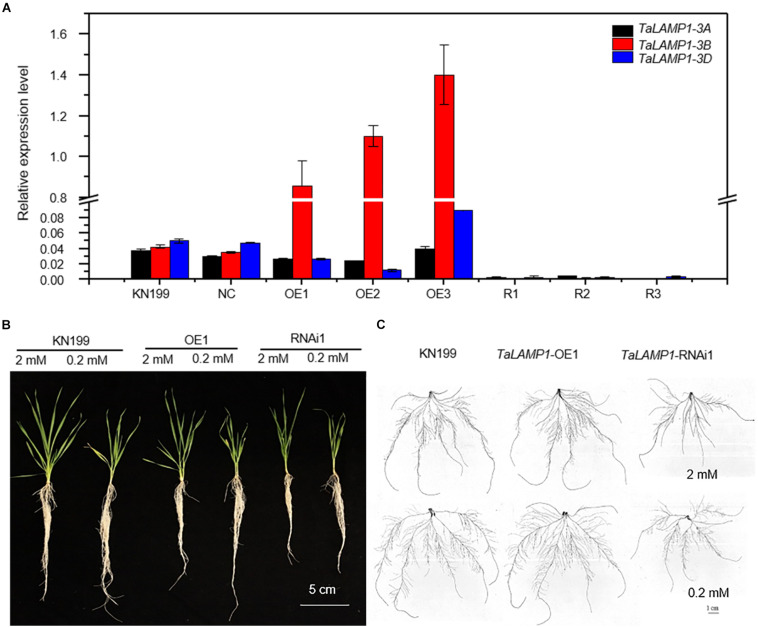
Phenotypes of the wild type and *TaLAMP1* transgenic lines under HN and LN conditions at the seedling stage. Seedlings of KN199 and *TaLAMP1* transgenic lines were grown for 12 days in the nutrient solution containing 2.0-mM NO_3_^–^ (High N treatment) and 0.2-mM NO_3_^–^ (Low N treatment); then, roots and shoots were collected for further analysis. KN199 and NC are wild types and negative transgenic lines, respectively. OE1, OE2, and OE3 are *TaLAMP1-3B* overexpression transgenic lines; R1, R2, and R3 are *TaLAMP1* knockdown transgenic lines. **(A)** Relative expression of *TaLAMP1* in the *TaLAMP1-3B* overexpression and *TaLAMP1* knockdown transgenic lines of wheat. Root samples were collected to evaluate expression level of *TaLAMP1*. Gene expression was normalized using expression of *TaActin*. Data are mean ± SE (*n* = 3). **(B,C)** Image of wheat plants **(B)** and root morphology **(C)**. 2 mM and 0.2 mM indicated N supply level. Bars = 5 cm.

The TaLAMP1s shared 95% amino acid sequence similarity between each other; they also have similar expression pattern during the developmental stage and in different N supply condition, so we chose *TaLAMP1-3B* as a representative gene to investigate the biological function; we developed *TaLAMP1-3B* overexpression and *TaLAMP1* RNAi lines by using the wheat variety KN199 as WT. Comparing with WT and negative control (NC) transgenic plants, the expression of *TaLAMP1-3B* was increased in the overexpression lines, and *TaLAMP1-3A*, *3B*, and *3D* were all downregulated in knockdown lines (Figure 3A). These results indicated that *TaLAMP1-3B* was successfully overexpressed in these three overexpression lines, and *TaLAMP1* was successfully knocked down in these three RNAi lines.

**FIGURE 4:**
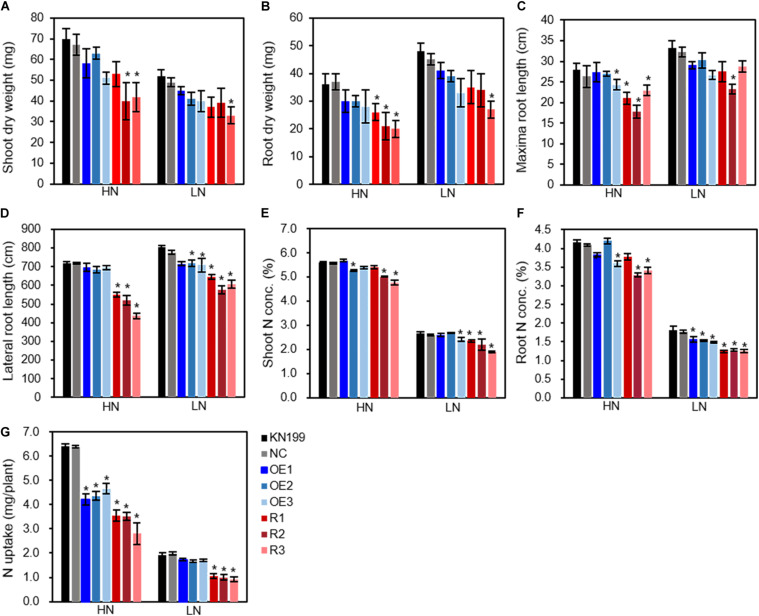
Growth and N-use-related traits of the wild type and *TaLAMP1* transgenic at seedling stage. Seedlings of KN199 and *TaLAMP1* transgenic lines were grown for 12 days in nutrient solution containing 2.0-mM NO_3_^–^ (High N treatment, HN) and 0.2-mM NO_3_^–^ (Low N treatment, LN); then, roots and shoots were collected for data analysis. KN199 and NC are wild types and negative transgenic lines, respectively. OE1, OE2, and OE3 indicated *TaLAMP1-3B* overexpression transgenic lines; R1, R2, and R3 indicated *TaLAMP1* knockdown transgenic lines. Data are means ± SE of four biological replicates. NC indicated mean value of negative transgenic lines separated from overexpression and knockdown lines. **(A)** Shoot dry weight; **(B)** Root dry weight; **(C)** Maxima root length; **(D)** Lateral root length; **(E)** Shoot N concentration; **(F)** Root N concentration; **(G)** N uptake. “*” indicates statistically significant differences between KN199 and transgenic lines at the *P* < 0.05 level.

We investigated growth and N-use-related traits of wheat seedlings grown in nutrient solutions containing 2-mM N (high N treatment) and 0.2-mM N (low N treatment). Compared with high N treatment, the seedling growth of wheat plants was significantly inhibited by low N treatment (Figures 3B,C). Compared with WT and NC, the transgenic lines tended to have lower trait values of shoot dry weight (Figure 4A), root dry weight (Figure 4B), maximum root length (Figure 4C), lateral root length (LRL, Figure 4D), shoot N concentration (Figure 4E), root N concentration (Figure 4F), and N uptake (Figure 4G), although the significant difference between the transgenic lines and WT depended on traits and N treatments. The data in Figure 4 also show that altering *TaLAMP1* expression changed the response of LRL and N uptake to N availability. Compared with high N treatment, low N increased LRL of WT by 12.0% while not increased that of overexpression lines (Figure 4D). We used the data in Figure 4G to calculate the response of N uptake to N availability by using the fold change of high N treatment vs. low N treatment. The average fold change (N uptake ratio of HN/LN) of WT, NC, the three overexpression lines, and the three knockdown lines were 3.32, 3.21, 2.59, and 3.33, respectively. These results indicated that altering *TaLAMP1* expression changed root morphology, and overexpressing *TaLAMP1* inhibited low-N-induced root branching and the response of N uptake to N availability.

**FIGURE 5:**
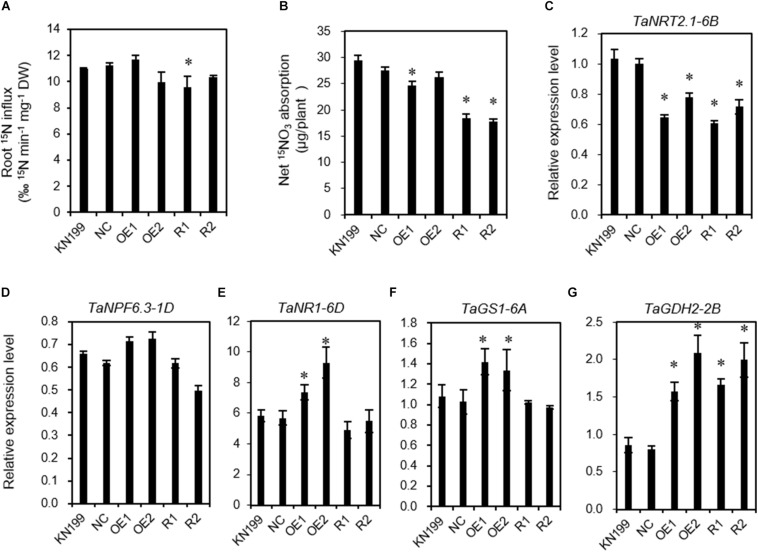
^15^NO_3_^–^ influx rate and gene expression of *TaLAMP1* transgenic seedlings. Seedlings of KN199 and *TaLAMP1* transgenic lines were grown for 7 days in the nutrient solution containing 2.0-mM NO3^–^ (HN), then transferred to 0.2-mM NO3^–^ (LN) for 2 days and resupply to 2.0-mM ^15^NO_3_^–^ for 5 min; roots were collected for ^15^N analysis and gene expression analysis. KN199 and NC are wild types and negative transgenic lines, respectively. OE1 and OE2 are *TaLAMP1-3B* overexpression transgenic lines; R1 and R2 are *TaLAMP1* knockdown transgenic lines. **(A,B)** Root ^15^NO_3_^–^ influx rate **(A)** and net ^15^N absorption in roots **(B)** of *TaLAMP1* transgenic lines. Data are shown as mean ± SE (*n* = 5). **(C–G)** Relative expression of *TaNRT2.1-6B* **(C)**, *TaNPF6.3-1D* **(D)**, *TaNR1-6D* **(E)**, *TaGS1-6A* **(F)**, and *TaGDH2-2B* **(G)**. Gene expression was normalized using the expression of *TaActin*. Data are the mean ± SE (*n* = 3). “^∗^” indicates statistically significant differences between KN199 and the transgenic lines at the *P* < 0.05 level.

Because the transgenic lines had lower N uptake than WT, we next examined root ^15^NO_3_^–^ influx rate. When the ^15^NO_3_^–^ influx rates were measured in 2-mM ^15^NO_3_^–^, one (OE2) of the two tested overexpression lines and both (R1 and R2) of the two knockdown lines had lower influx rates than did WT and NC, but only R1 showed a significant difference with WT and NC (Figure 5A). All the tested transgenic lines exhibited lower total ^15^N uptake than WT and NC (Figure 5B). We also analyzed the expression of genes involved in N uptake and assimilation. Compared with the WT and negative control plants, the overexpression lines displayed lower expression of the nitrate transporter *TaNRT2.1-6B* (Figure 5C) and higher expression of the nitrate transporter *TaNFP6.3-1D* (Figure 5D), nitrate reductase *TaNR1-6D* (Figure 5E), and glutamate dehydrogenase *TaGDH2-2B* (Figure 5G). The knockdown lines had lower expression of *TaNRT2.1-6B* (Figure 5C) and higher expression of *TaGDH2-2B* (Figure 5G). There was a similar expression level of the glutamine synthetase *TaGS1-6A* between *TaLMAP1-3B* transgenic lines and WT (Figure 5F).

### Agronomic Traits of *TaLAMP1* Transgenic Lines in the Field Experiment

**FIGURE 6:**
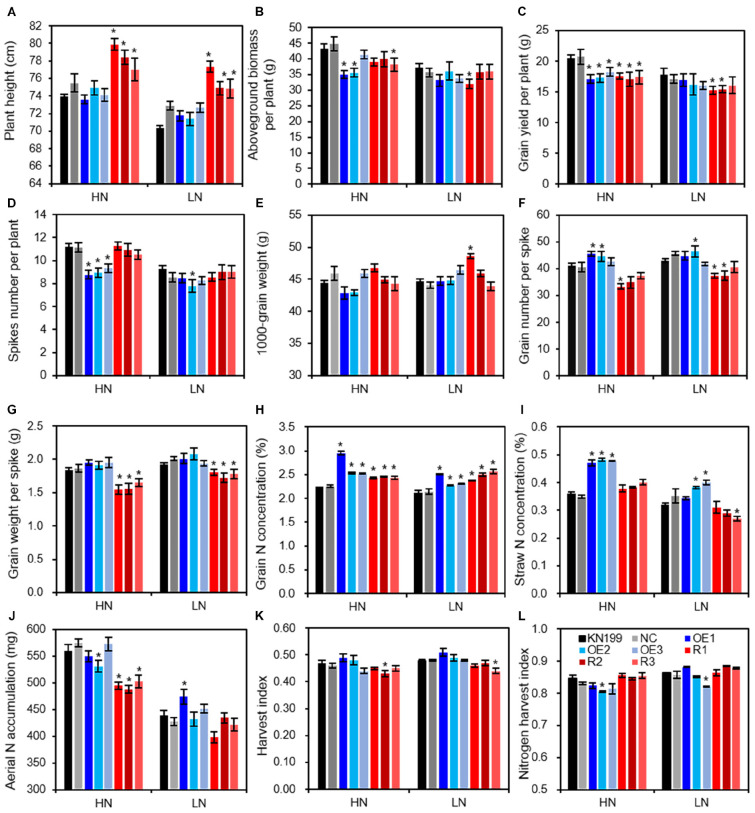
Agronomic and N-use-related traits of the wide type KN199 and *TaLAMP1* transgenic in field experiment in 2015–2016 growing season. **(A)** Plant height; **(B)** Biomass per plant; **(C)** Grain yield per plant; **(D)** Spikes number per plant; **(E)** 1,000-grain weight; **(F)** Grain number per spike; **(G)** Grain weight per spike; **(H)** Grain N concentration; **(I)** Straw N concentration; **(J)** Aerial N accumulation; **(K)** Harvest index; **(L)** Nitrogen harvest index. KN199 and NC are wild types and negative transgenic lines, respectively. OE1, OE2, and OE3 are *TaLAMP1-3B* overexpression transgenic lines; R1, R2, and R3 are *TaLAMP1* knockdown transgenic lines. HN represents the normal N condition (225 kg N fertilizer per ha) for wheat growth in the field. LN represents low N treatment (94.5 kg N fertilizer per ha) for wheat growth in the field. Data are the mean ± SE of four replications, each with 30 plants. “*” indicates statistically significant differences between KN199 and the transgenic lines at the *P* < 0.05 level.

In the field experiment, we investigated the growth performance and agronomic traits under low N fertilization (94.5 kg N fertilizer per ha) and high N fertilization (225 kg N fertilizer per ha) in the 2015–2016 growing season by using the T4 transgenic lines. Under high N conditions, at least two of the three overexpression lines had lower biomass yield (Figure 6B), grain yield (Figure 6C), and spike number (Figure 6D) but had higher grain number per spike (Figure 6F) than WT; there was no significant difference between the overexpression lines and WT in plant height (Figure 6A), 1,000-grain weight (TGW, Figure 6E), and harvest index (HI, Figure 6K). These results indicated that overexpressing *TaLAMP1-3B* reduced biomass and grain yield mainly by reducing spike number. The knockdown lines had higher plant height (Figure 6A) but lower grain yield (Figure 6C) and grain weight per spike (Figure 6G) than WT. There was no significant difference between the knockdown lines and WT in spike number per plant (Figure 6D) and TGW (Figure 6E). These results indicated that the knockdown of *TaLAMP1* reduced grain yield mainly by reducing grain weight per spike. Under low N conditions, the overexpression lines showed no difference with WT in most investigated agronomic traits (Figures 6A–G,K). At least two of the knockdown lines had higher plant height (Figure 6A) but lower grain yield (Figure 6C), grain number per spike (Figure 6F), and grain weight per spike (Figure 6G) than WT. These results indicated that altering *TaLAMP1* expression changed plant architecture reflecting by plant height, spike number per plant, and grain number per spike, and the phenotypes of the agronomic traits in the transgenic lines depended on N supply levels. N fertilizer apparently increased grain yield in WT (Figure 6C). However, the increasing effects were higher in WT than in the transgenic lines. Analyzing the response of yield components to N fertilizer revealed that the poor yield response of the overexpression lines was mainly due to the poor response of spike number to N fertilizer, whereas that of the knockdown lines has mainly resulted from the poor response of grain weight per spike (a combination effect of grain number per spike and TGW) to N fertilizer (Table 1).

**TABLE 1:** Response of yield traits to N fertilizer of the *TaLAMP1* transgenic lines.

	Grain yield per plant (%)	Spike number (%)	Grain weight per spike (%)	1,000-grain weight (%)	Grain number per spike (%)
KN199	17.830.43	20.340.27	−4.170.11	−0.560.05	−4.040.15
Negative control	20.960.31	23.90.14	−6.460.47	1.110.42	−5.250.47
OE1	1.120.09*	3.660.25*	−2.990.29	−4.270.31	1.880.11
OE2	6.410.11*	13.420.06	−8.171.11	−4.280.14	−4.111.65
OE3	13.580.16	12.670.03	−1.210.49	−1.140.46	2.040.24
R1	12.661.15	31.810.18	−14.360.11*	−3.870.18	−11.090.25*
R2	10.820.22*	20.930.26	−9.30.17*	−2.160.62	−6.720.44
R3	8.210.71*	16.520.02	−7.31.22	0.980.09	−8.031.81

KN199 and NC are wild types and negative transgenic lines, respectively. OE1, OE2, and OE3 are *TaLAMP1-3B* overexpression transgenic lines; R1, R2, and R3 are *TaLAMP1* knockdown transgenic lines. N fertilizer response value means the value of (HN-LN)/LN × 100%. “*” indicates statistically significant differences between KN199 and the transgenic lines at the *P* < 0.05 level.

### N Use-Related Traits of *TaLAMP1* Transgenic Lines in Field Experiment

**FIGURE 7:**
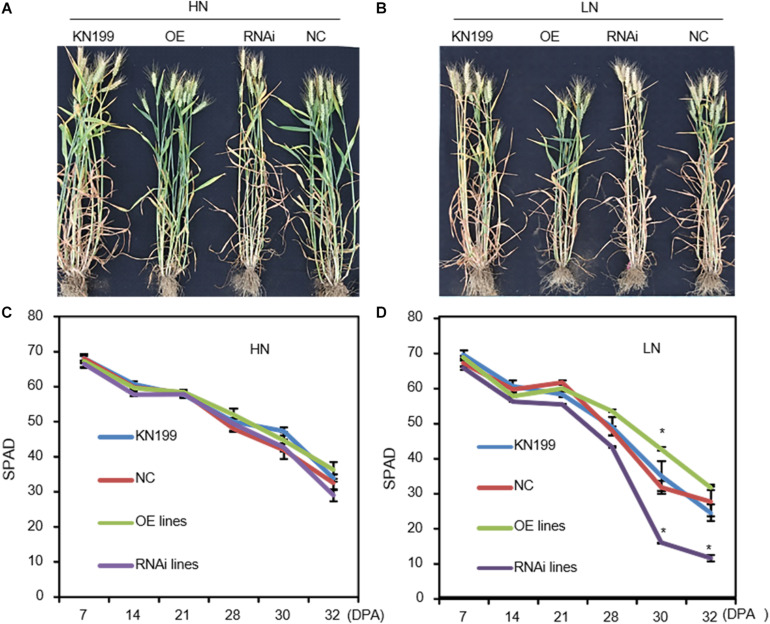
SPAD values in the flag leaves of the *TaLAMP1* transgenic lines and wild type after flowering in 2015–2016 field experiment. **(A,B)** Images of the transgenic lines and wild-type plants under high N **(A)** and low N **(B)** conditions at 4 weeks after flowering. **(C,D)** SPAD values in the flag leaves under high N **(C)** and low N **(D)** conditions. Data are mean ± SE of four biological replications. * indicates a significant difference between the transgenic lines and control plants (wild type and negative control) at *P* < 0.05.

The transgenic lines did not show visible leaf color difference with WT and NC at anthesis (Zadoks stage 64). After anthesis (Zadoks stage 77), the overexpression lines of *TaLAMP1* showed a certain degree of stay-green, whereas the knockdown lines exhibited faster chlorophyll degradation than WT, especially under low N conditions (Figures 7A,B). For instance, SPAD value in the flag leaves of knockdown lines was much lower than in WT after 21 days post-anthesis (Zadoks stage 85) under low N condition, demonstrating that chlorophyll degradation proceeded at a significantly more rapid pace in the knockdown lines than WT (Figures 7C,D).

We measured N concentration in grains and straws and calculated aerial N accumulation (ANA) and N harvest index at maturity. The transgenic lines had higher grain N concentration (GNC) than WT under both low and high N conditions (Figure 6H). The overexpression lines had higher straw N concentration than WT under high N conditions (Figure 6I). The knockdown lines had lower ANA than WT under high N conditions (Figure 6J). The transgenic lines showed similar values in HI and N harvest index (Figures 6K,L) with WT under both low and high N conditions.

## Discussion

### *TaLAMP1* Responded to N Availability

Our microarray analysis identified two N-responsive probe sets of *TaLAMP1* genes, which belong to a conserved superfamily of GCPII. The qRT-PCR further confirmed that *TaLAMP1-3A*, *-3B*, and *-3D* were upregulated in both shoots and roots by low N availability (Figures 1A,B). The altered expression of genes involved in N uptake and assimilation in the transgenic lines suggests a possible role of *TaLAMP1* in mediating N metabolism. The contents of amino acids were changed in *Arabidopsis amp1* mutant, suggesting that AMP1 may involve in the amino acid metabolism in a plant (Shi et al., 2013). N fertilizer is well known to increase plant cytokinin biosynthesis through upregulating *isopentenyltransferase* (*IPT*) genes (Gu et al., 2018). An increased cytokinin level was observed in the *Arabidopsis amp1* mutant (Chaudhury et al., 1993; Helliwell et al., 2001) and the rice *pla3* mutant (Kawakatsu et al., 2009). We also observed that the ZR level in whole seedlings of the knockdown lines was higher than did WT (Supplementary Figure 4). As such, AMP1 homologs have a conserved role in regulating cytokinin homeostasis in higher plants. The increased ZR level in the *TaLAMP1*-RNAi lines suggests that the downregulation of *TaLAMP1* by high N availability may be critical for the response of cytokinin biosynthesis to N fertilizer.

### *TaLAMP1* Regulates N-Use-Related Traits

Both the overexpression and knockdown lines of *TaLAMP1* had lower N uptake than WT under at seedling stage when the plants were grown in hydroponic culture (Figure 4G). The knockdown lines also had lower ANA under high N conditions in the field experiment (Figure 6J). A smaller root size reflected by lower root dry weight (Figure 4B), shorter maximum root length (Figure 4C), and lateral root length (LRL, Figure 4D) may contribute to the lower N uptake of the transgenic lines. The lower N uptake of the knockdown lines may also be associated with the lower nitrate influx rate and expression of *TaNRT2.1-6B* in roots (Figures 5A,C).

Altering the expression of *TaLAMP1* also greatly changed N concentrations in roots and aerial parts at the seedling stage (hydroponic culture) and maturity (field experiment). Both overexpression and knockdown of *TaLAMP1* tended to reduce the N concentrations in shoots and roots at the seedling stage, regardless of N supply levels (Figures 4E,F). However, both the overexpression and knockdown lines of *TaLAMP1* had significantly higher GNC than WT under high and low N conditions (Figure 6H). The distinct effects of *TaLAMP1* on N concentrations may be due to the different growth conditions (hydroponic culture vs. field experiment) or developing stages (seedling stage vs. maturity). Another possible explanation for these distinct effects is that *TaLAMP1* may have roles in mediating post-anthesis N uptake or N remobilization. Grain N accumulation is the sum of N uptake and remobilization from the vegetative parts post-anthesis (Pan et al., 2006), and post-anthesis N uptake and N remobilization positively contribute to grain N accumulation in wheat (Kichey et al., 2007). The accelerated leaf senescence in the knockdown lines under low N conditions supported the role of *TaLAMP1* in N remobilization. There was a certain negative correlation between grain yield and grain protein concentration (Kibite and Evans, 1984; Bogard et al., 2010), and low input of N fertilizer can reduce both grain yield and grain protein concentration. As overexpressing *TaLAMP1-3B* greatly increased GNC (7.1–18.4% higher than WT) but not significantly reduced grain yield under low N conditions (Figures 6C,H), *TaLAMP1-3B* is therefore valuable in engineering wheat for low input agriculture.

### *TaLAMP1* Influences Plant Architecture and Yield Response to N Fertilizer

The data collected from the hydroponic culture (Figure 4) and the field experiment (Figure 6) showed the roles of *TaLAPM1* not only in regulating root morphology but also in shaping plant architecture (plant height, spike number, and grain number). However, the phenotypes of the transgenic lines depended on N availability, and the transgenic line and WT differed in response to N fertilizer. At the seedling stage, overexpressing *TaLAMP1-3B* inhibited low-N-induced root branching and the response of N uptake to N availability (Figures 4D,G). In the field experiment, overexpressing *TaLAMP1* significantly inhibited the response of grain yield to N fertilizer, mainly by inhibiting the response of spike number per plant to N fertilizer (Table 1). Considering the downregulation of *TaLAMP1* expression by high N treatment and inhibitory effect of overexpressing *TaLAMP1* on spike number per plant, it is assumed that the downregulation of *TaLAMP1* expression by high N treatment is required for spike number response to N fertilizer and, hence, the yield response to N fertilizer.

*ZmVP8* in maize and *OsPLA3* in rice have been shown to regulate plant height, and loss of function of these two genes resulted in shortened internodes phenotype (Kawakatsu et al., 2009; Lv et al., 2014). Our current study showed that the knockdown lines of *TaLAMP1* had higher plant height than WT (Figure 6A). Phylogenetic analysis of GCPII proteins found that ZmVP8 and OsPLA3 displayed the closest relation with AtAMP1, whereas the three TaLAMP1s did not belong to the clade that contains AtAMP1, ZmVP8, and OsPLA3 (Figure 2). As such, *AMP1* (*ZmVP8* and *OsPLA3*) and *AMP1*-like (*TaLAMP1*) genes differentially regulate plant height of cereal crops, although the rice *pla3* mutant and the *TaLAMP1* knockdown lines had an increased cytokinin level. The increased ZR level could partially explain the increased plant height of the knockdown lines.

The increased ZR level in the knockdown lines may not be associated with the reduced grain number per plant in the knockdown lines, as it has been reported that increased cytokinin levels benefit more grain numbers per spike in rice (Ashikari et al., 2005). A recent study showed that the *Arabidopsis amp1* mutant has extra stem cell niches in the presence of an intact primary shoot apical meristem, and this defect appears to be neither a specific consequence of the increased cytokinin levels in *amp1* mutant nor directly mediated by the WUSCHEL/CLAVATA feedback loop (Huang et al., 2015). The underlying mechanism for the altered root morphology and plant architecture of the *TaLAMP1* transgenic lines is still needed for further investigation. Although we observed the changes in N concentration in the investigated organs of the transgenic lines, we did not know the roles of *TaLAMP1* in regulating the distribution of N metabolites in specific cells. It has been reported that the loss-off function of *AMP1* in *Arabidopsis* exerts a large influence on amino acid composition (Shi et al., 2013). Our current study showed that the transgenic lines had altered expression of genes involved in N assimilations (Figures 5D–G). Dissecting the roles of *TaLAMP1* in regulating N assimilation and distribution of N metabolites will help us understand the altered root morphology and plant architecture in the transgenic lines. In *Arabidopsis thaliana*, miRNAs are mainly loaded into ARGONAUTE1 (AGO1) to posttranscriptionally regulate gene expression; AMP1 and ARGONAUTE1 are colocalized in the endoplasmic reticulum, which implicates the endoplasmic reticulum in miRNA-mediated gene silencing; a possible role of TaLAMP1 is to influence the expression of nitrogen-related miRNAs and their target genes by AGO1 complex the molecular processes are still unknown; further research is needed to investigate the upstream regulatory genes of *TaLAMP1*; it will help us to know the function and expression in the pathway of nitrogen absorption and accumulation.

## Data Availability Statement

The datasets presented in this study can be found in online repositories. The names of the repository/repositories and accession number(s) can be found in the article/Supplementary Material.

## Author Contributions

YT and JS designed the experiments and wrote the manuscript. JS performed most of the experiments and analyzed the data. Both authors contributed to the article and approved the submitted version.

## Conflict of Interest

The authors declare that the research was conducted in the absence of any commercial or financial relationships that could be construed as a potential conflict of interest.

## Footnotes

1 www.zeiss.com

2 http://www.gramene.org/
