# Supplementary material for: TaLAMP1 Plays Key Roles in Plant Architecture and Yield Response to Nitrogen Fertilizer in Wheat
Source: Front Plant Sci. 2021 Jan 8;11:598015. doi: 10.3389/fpls.2020.598015 (PMC7832495; doi:10.3389/fpls.2020.598015)
Supplement: Supplementary file 1 [file Presentation_1.PPTX]

## Slide 1
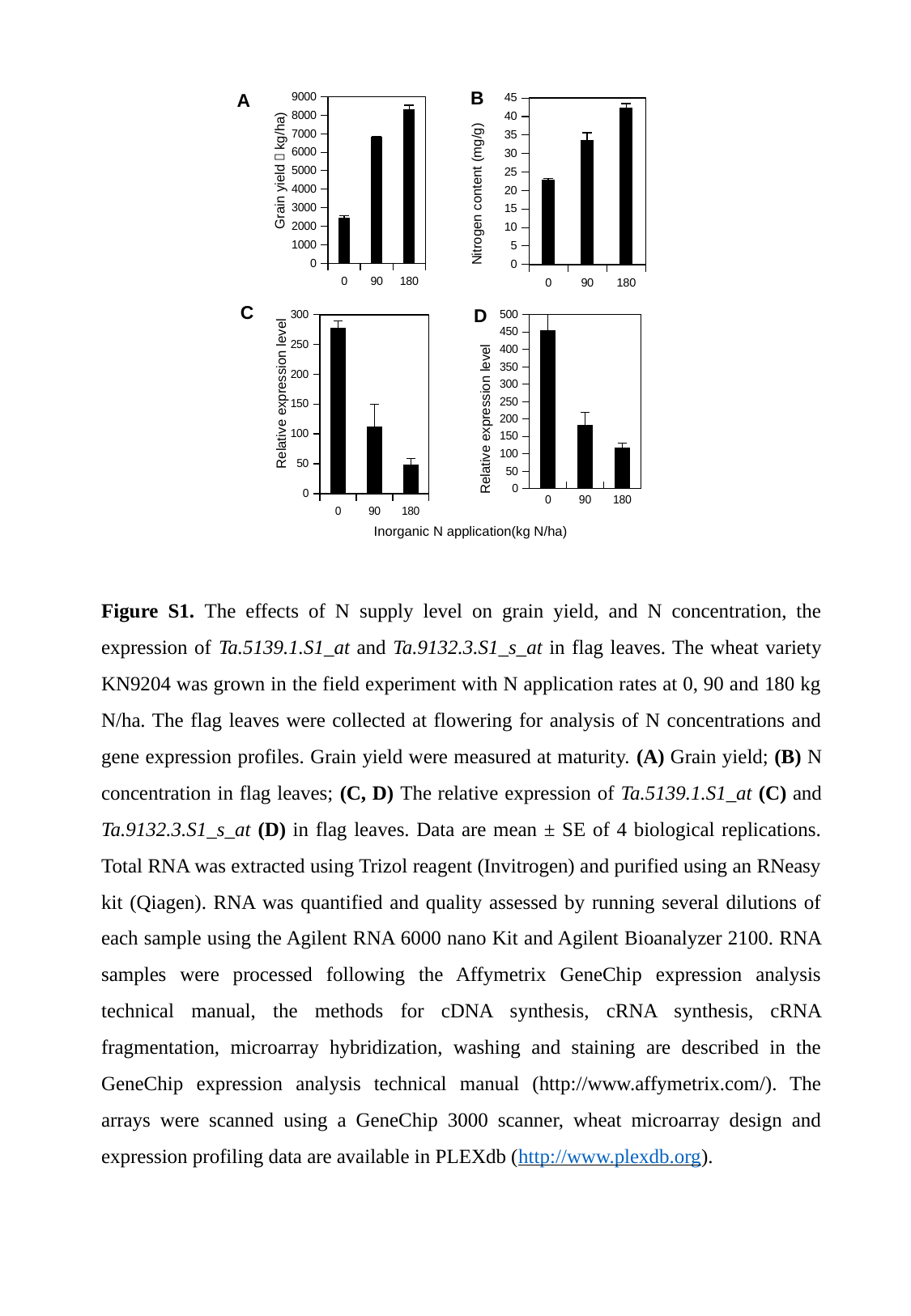

B
A
### Chart
| Category | KN9204 |
|---|---|
| 0 | 2430.0 |
| 90 | 6808.333333333333 |
| 180 | 8299.444444444445 |
### Chart
| Category | KN9204 |
|---|---|
| 0 | 22.766512149257224 |
| 90 | 33.57827822217187 |
| 180 | 42.32251095682673 |Grain yield（kg/ha)
Nitrogen content (mg/g)
C
D
### Chart
| Category | KN9204 |
|---|---|
| 0 | 276.8764857333333 |
| 90 | 111.09211195666667 |
| 180 | 47.76483400666667 |
### Chart
| Category | KN9204 |
|---|---|
| 0 | 453.9610638666666 |
| 90 | 182.58485069999998 |
| 180 | 116.53392132333333 |Relative expression level
Inorganic N application(kg N/ha)
Relative expression level
Figure S1. The effects of N supply level on grain yield, and N concentration, the expression of Ta.5139.1.S1_at and Ta.9132.3.S1_s_at in flag leaves. The wheat variety KN9204 was grown in the field experiment with N application rates at 0, 90 and 180 kg N/ha. The flag leaves were collected at flowering for analysis of N concentrations and gene expression profiles. Grain yield were measured at maturity. (A) Grain yield; (B) N concentration in flag leaves; (C, D) The relative expression of Ta.5139.1.S1_at (C) and Ta.9132.3.S1_s_at (D) in flag leaves. Data are mean ± SE of 4 biological replications. Total RNA was extracted using Trizol reagent (Invitrogen) and purified using an RNeasy kit (Qiagen). RNA was quantified and quality assessed by running several dilutions of each sample using the Agilent RNA 6000 nano Kit and Agilent Bioanalyzer 2100. RNA samples were processed following the Affymetrix GeneChip expression analysis technical manual, the methods for cDNA synthesis, cRNA synthesis, cRNA fragmentation, microarray hybridization, washing and staining are described in the GeneChip expression analysis technical manual (http://www.affymetrix.com/). The arrays were scanned using a GeneChip 3000 scanner, wheat microarray design and expression profiling data are available in PLEXdb (http://www.plexdb.org).

## Slide 2
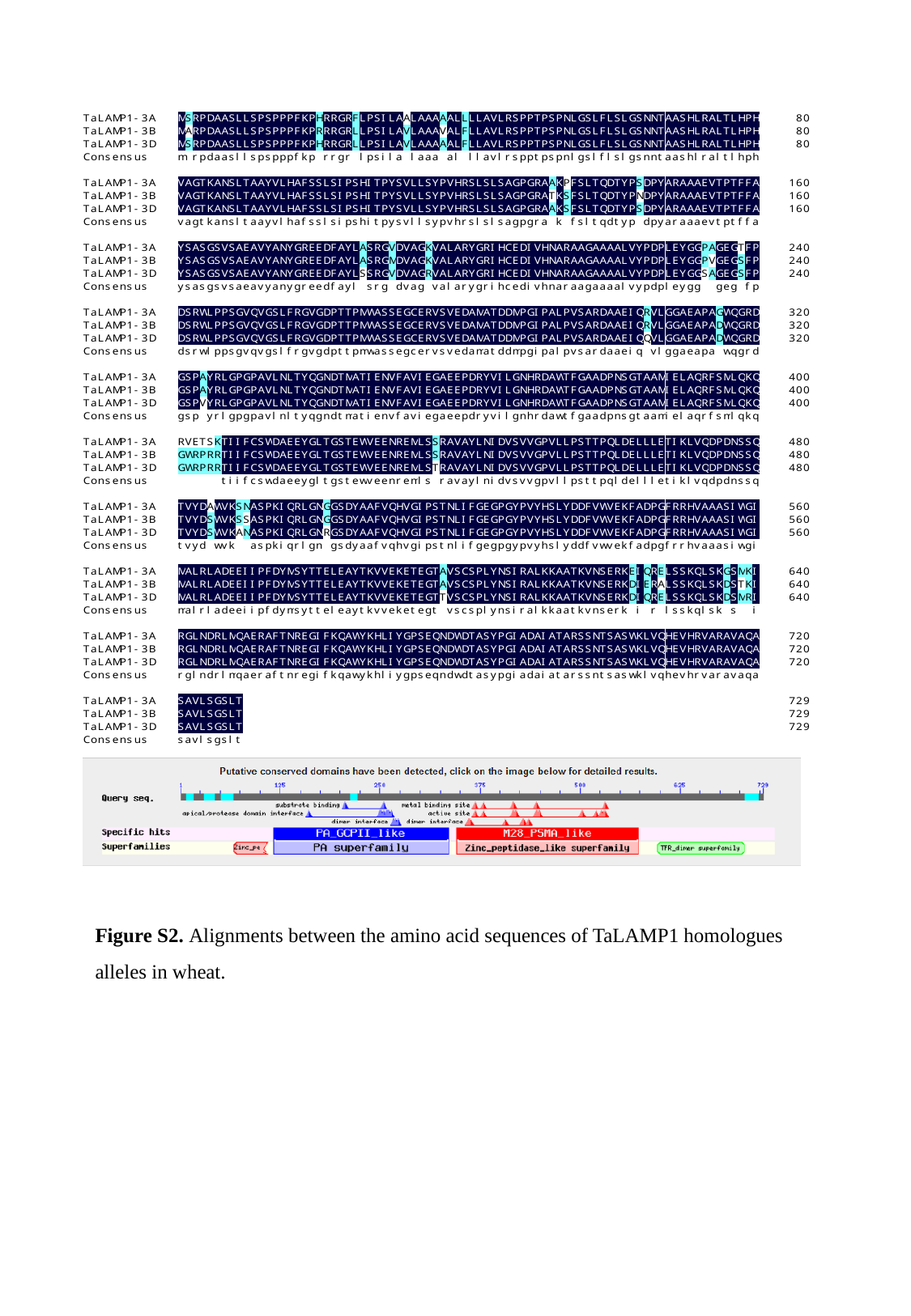

Figure S2. Alignments between the amino acid sequences of TaLAMP1 homologues alleles in wheat.

## Slide 3
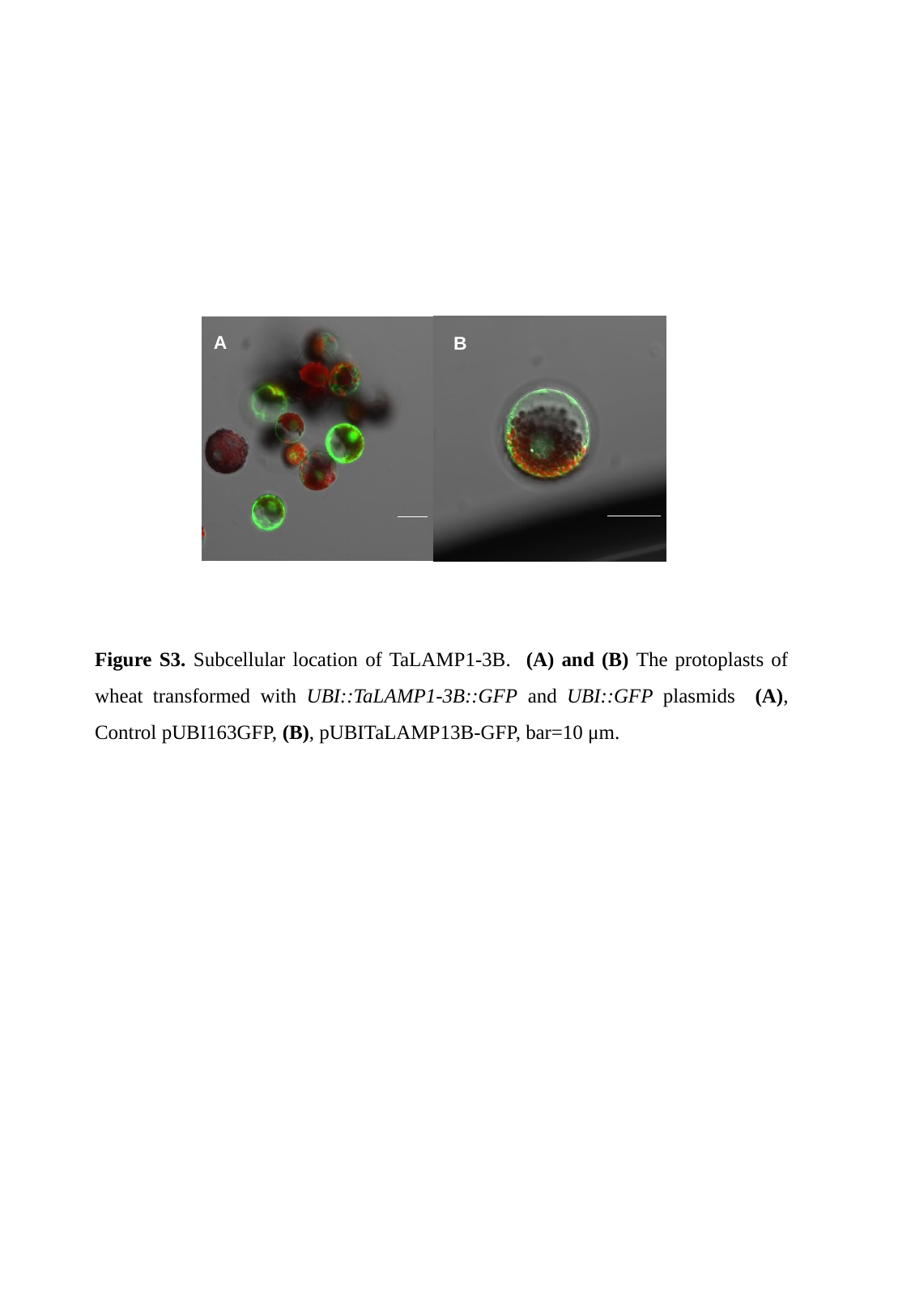

B
A
B
Figure S3. Subcellular location of TaLAMP1-3B. (A) and (B) The protoplasts of wheat transformed with UBI::TaLAMP1-3B::GFP and UBI::GFP plasmids (A), Control pUBI163GFP, (B), pUBITaLAMP13B-GFP, bar=10 μm.

## Slide 4
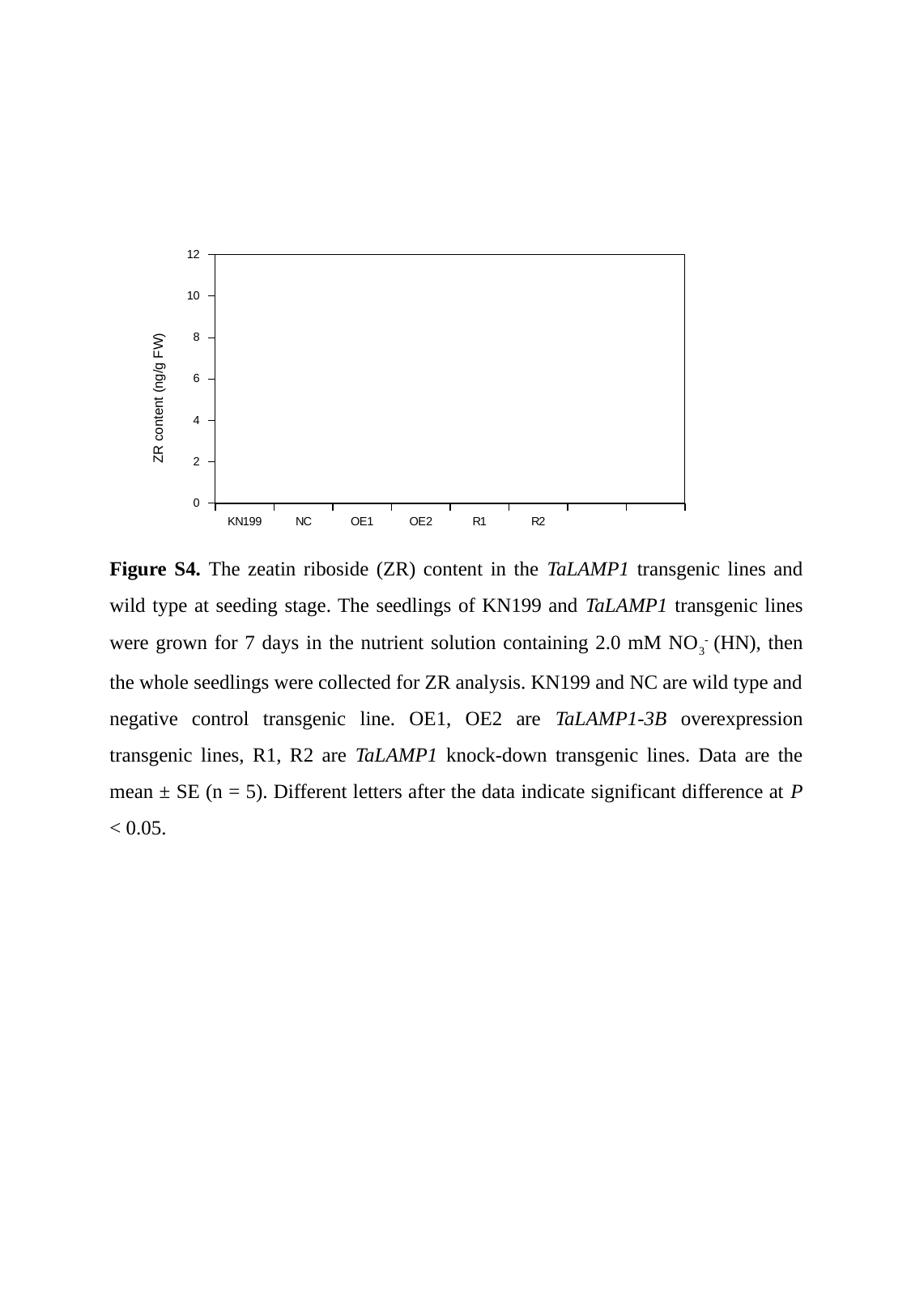

### Chart
| Category | |
|---|---|
| KN199 | 6.14 |
| NC | 6.016666666666666 |
| OE1 | 6.736666666666667 |
| OE2 | 6.12666666666667 |
| R1 | 8.056666666666667 |
| R2 | 10.003333333333334 |ZR content (ng/g FW)
Figure S4. The zeatin riboside (ZR) content in the TaLAMP1 transgenic lines and wild type at seeding stage. The seedlings of KN199 and TaLAMP1 transgenic lines were grown for 7 days in the nutrient solution containing 2.0 mM NO3- (HN), then the whole seedlings were collected for ZR analysis. KN199 and NC are wild type and negative control transgenic line. OE1, OE2 are TaLAMP1-3B overexpression transgenic lines, R1, R2 are TaLAMP1 knock-down transgenic lines. Data are the mean ± SE (n = 5). Different letters after the data indicate significant difference at P < 0.05.

## Slide 5
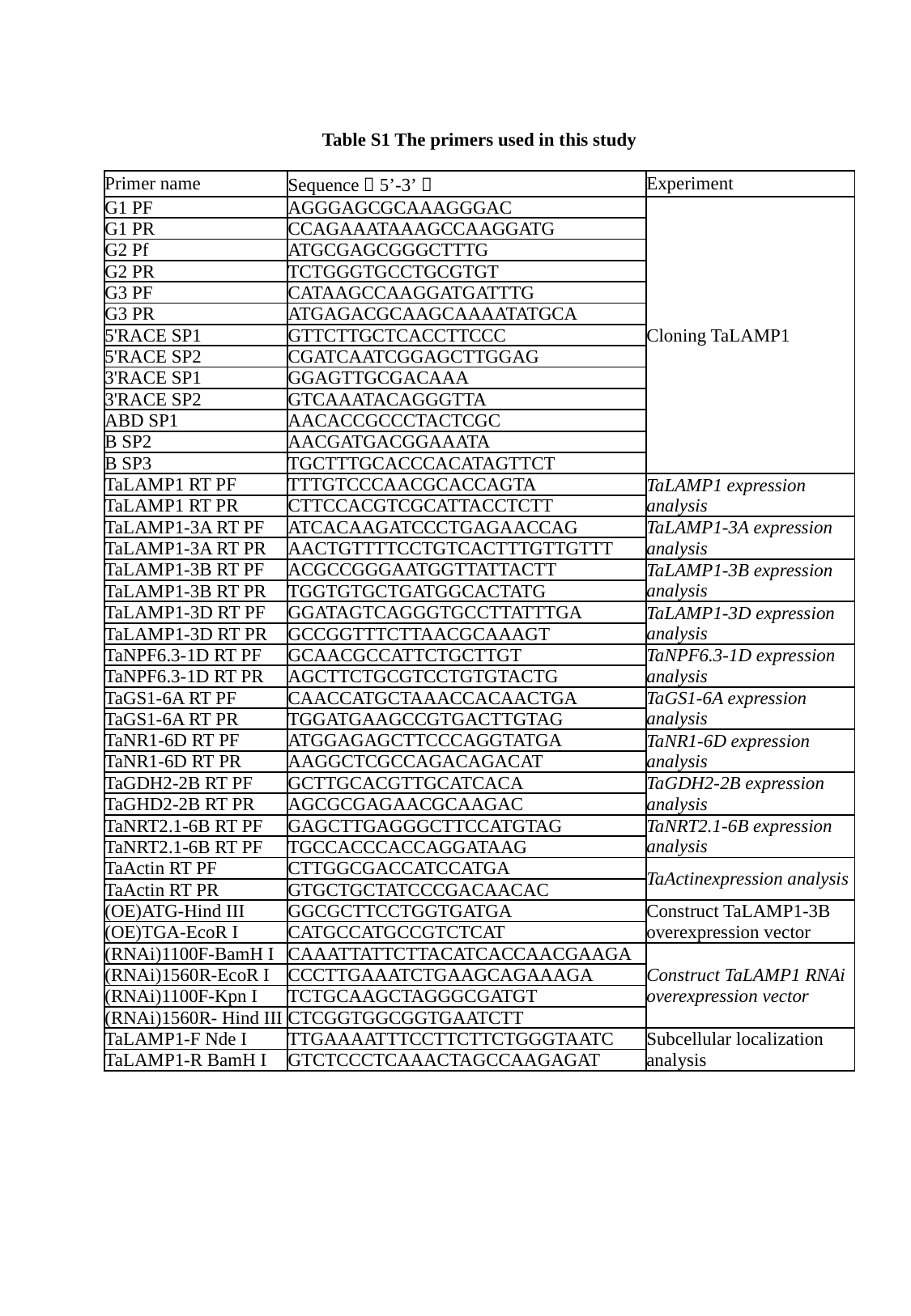

| Table S1 The primers used in this study | | |
| --- | --- | --- |
| Primer name | Sequence（5’-3’） | Experiment |
| G1 PF | AGGGAGCGCAAAGGGAC | Cloning TaLAMP1 |
| G1 PR | CCAGAAATAAAGCCAAGGATG | |
| G2 Pf | ATGCGAGCGGGCTTTG | |
| G2 PR | TCTGGGTGCCTGCGTGT | |
| G3 PF | CATAAGCCAAGGATGATTTG | |
| G3 PR | ATGAGACGCAAGCAAAATATGCA | |
| 5'RACE SP1 | GTTCTTGCTCACCTTCCC | |
| 5'RACE SP2 | CGATCAATCGGAGCTTGGAG | |
| 3'RACE SP1 | GGAGTTGCGACAAA | |
| 3'RACE SP2 | GTCAAATACAGGGTTA | |
| ABD SP1 | AACACCGCCCTACTCGC | |
| B SP2 | AACGATGACGGAAATA | |
| B SP3 | TGCTTTGCACCCACATAGTTCT | |
| TaLAMP1 RT PF | TTTGTCCCAACGCACCAGTA | TaLAMP1 expression analysis |
| TaLAMP1 RT PR | CTTCCACGTCGCATTACCTCTT | |
| TaLAMP1-3A RT PF | ATCACAAGATCCCTGAGAACCAG | TaLAMP1-3A expression analysis |
| TaLAMP1-3A RT PR | AACTGTTTTCCTGTCACTTTGTTGTTT | |
| TaLAMP1-3B RT PF | ACGCCGGGAATGGTTATTACTT | TaLAMP1-3B expression analysis |
| TaLAMP1-3B RT PR | TGGTGTGCTGATGGCACTATG | |
| TaLAMP1-3D RT PF | GGATAGTCAGGGTGCCTTATTTGA | TaLAMP1-3D expression analysis |
| TaLAMP1-3D RT PR | GCCGGTTTCTTAACGCAAAGT | |
| TaNPF6.3-1D RT PF | GCAACGCCATTCTGCTTGT | TaNPF6.3-1D expression analysis |
| TaNPF6.3-1D RT PR | AGCTTCTGCGTCCTGTGTACTG | |
| TaGS1-6A RT PF | CAACCATGCTAAACCACAACTGA | TaGS1-6A expression analysis |
| TaGS1-6A RT PR | TGGATGAAGCCGTGACTTGTAG | |
| TaNR1-6D RT PF | ATGGAGAGCTTCCCAGGTATGA | TaNR1-6D expression analysis |
| TaNR1-6D RT PR | AAGGCTCGCCAGACAGACAT | |
| TaGDH2-2B RT PF | GCTTGCACGTTGCATCACA | TaGDH2-2B expression analysis |
| TaGHD2-2B RT PR | AGCGCGAGAACGCAAGAC | |
| TaNRT2.1-6B RT PF | GAGCTTGAGGGCTTCCATGTAG | TaNRT2.1-6B expression analysis |
| TaNRT2.1-6B RT PF | TGCCACCCACCAGGATAAG | |
| TaActin RT PF | CTTGGCGACCATCCATGA | TaActinexpression analysis |
| TaActin RT PR | GTGCTGCTATCCCGACAACAC | |
| (OE)ATG-Hind III | GGCGCTTCCTGGTGATGA | Construct TaLAMP1-3B overexpression vector |
| (OE)TGA-EcoR I | CATGCCATGCCGTCTCAT | |
| (RNAi)1100F-BamH I | CAAATTATTCTTACATCACCAACGAAGA | Construct TaLAMP1 RNAi overexpression vector |
| (RNAi)1560R-EcoR I | CCCTTGAAATCTGAAGCAGAAAGA | |
| (RNAi)1100F-Kpn I | TCTGCAAGCTAGGGCGATGT | |
| (RNAi)1560R- Hind III | CTCGGTGGCGGTGAATCTT | |
| TaLAMP1-F Nde I | TTGAAAATTTCCTTCTTCTGGGTAATC | Subcellular localization analysis |
| TaLAMP1-R BamH I | GTCTCCCTCAAACTAGCCAAGAGAT | |
